# Thermal‐Ionic Coupling in Transpiration‐Inspired Janus Nanofiber Membranes: Synergistic Dry‐Wet Interface Engineering for Solar‐Driven Electricity Generation

**DOI:** 10.1002/advs.77250

**Published:** 2026-08-17

**Authors:** Qin Su, Haidi Wu, Tingting Zheng, Di He, Huaiguo Xue, Longcheng Tang, Yongqian Shi, Xuejun Lai, Jiefeng Gao, Wancheng Gu, Jun Yan

**Affiliations:** ^1^ School of Chemistry and Materials Yangzhou University Yangzhou Jiangsu China; ^2^ Key Laboratory of Organosilicon Chemistry and Material Technology of MoE College of Material Chemistry and Chemical Engineering Hangzhou Normal University Hangzhou China; ^3^ College of Environment and Safety Engineering Fuzhou University Fuzhou China; ^4^ School of Materials Science and Engineering Key Laboratory of Guangdong Province for High Property and Functional Polymer Materials South China University of Technology Guangzhou China

**Keywords:** dry‐wet interface, electricity generation, interfacial evaporation, janus nanofiber membranes

## Abstract

Harnessing solar evaporation for simultaneous steam and electricity generation is an emerging technology for sustainable energy‐water systems, yet its development is fundamentally constrained by the inability to maintain a stable interfacial potential gradient in homogeneous materials. Here, we introduce a dry‐wet interface engineering strategy that enables persistent thermal‐ionic coupling for continuous electricity generation. Inspired by leaf transpiration, an asymmetric Janus nanofiber membrane is constructed to form a stable hydrophilic‐hydrophobic interface that simultaneously regulates fluid distribution and interfacial charge dynamics. This architecturally confined interface induces hydration‐triggered surface ionization on the hydrophilic layer, generating an intrinsic potential difference continuously reinforced by evaporation‐driven directional ion transport. As a result, the Janus evaporator delivers a stable open‐circuit voltage of 0.22 V under 1 sun illumination, 57% higher than natural evaporation, and exhibits scalable output up to 2.21 V via modular integration. Mechanistic investigations combining experiments and multiscale simulations reveal that the sustained electrical output arises from the synergistic coupling of interfacial potential modulation, ion‐selective transport, and localized photothermal heating. The dry‐wet interface plays a decisive role by stabilizing charge separation and suppressing potential dissipation. This work establishes a general framework for interfacial potential engineering in evaporation‐driven systems, enabling simultaneous solar desalination and electricity generation.

## Graphical abstract

A transpiration‐inspired Janus nanofiber membrane creates a persistent dry‐wet interface that couples localized solar heating with hydration‐induced ion regulation. The asymmetric architecture sustains directional water and ion transport, suppresses salt accumulation, and enables simultaneous seawater evaporation and stable electricity generation, offering a new strategy for integrated solar water‐energy conversion.

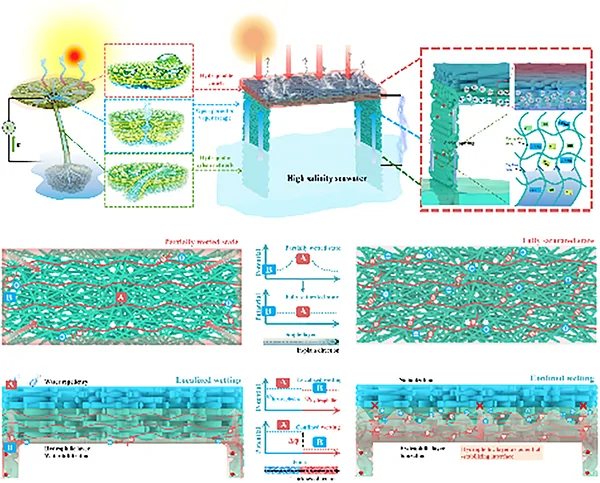

## 1 Introduction

The accelerating global crises of energy depletion and freshwater scarcity have brought into sharp focus the need for sustainable technologies capable of coupling energy harvesting with water purification [1]. Conventional energy conversion and desalination systems such as photovoltaics, thermoelectrics, and reverse osmosis typically operate independently and require substantial infrastructure and energy input [2]. In contrast, the interaction between water and functional solid interfaces provides an abundant yet underexplored energy source [3]. The emerging field of hydrovoltaic technology, which harvests electricity from water movement or ion diffusion across solid‐liquid interfaces, offers a green and ubiquitous approach to generate electricity from natural water dynamics [4]. The underlying mechanism involves the coupling of capillary‐driven flow, ion migration, and electric double layer (EDL) modulation, which together induce electrokinetic potentials along hydrated interfaces [5]. However, most reported hydrovoltaic systems exhibit limited output voltage, unstable performance, and weak control over interfacial ion transport, primarily due to inadequate design of surface chemistry and structure [6]. Overcoming these constraints requires the rational integration of materials with tailored wettability, chemical functionality, and charge‐regulation capability to sustain directional water and ion motion [7].

Meanwhile, solar‐driven interfacial evaporation (SDIE), a rapidly advancing strategy for photothermal desalination, offers an ideal physical platform to enhance electrokinetic energy conversion [8, 9]. By confining solar heat to the water‐air interface, photothermal materials induce localized vaporization while maintaining efficient water transport through interconnected capillary pathways [10, 11]. Importantly, the evaporation process inherently drives continuous water flow and ion redistribution, establishing dynamic concentration and potential gradients that can be harnessed for electricity generation [12, 13]. However, the interfacial complexity accompanying this process introduces substantial challenges. Salt accumulation at the evaporative surface can block water channels, hinder heat transfer, and distort local ion distributions, leading to a sharp decline in both evaporation efficiency and electrical output over time [14, 15]. In addition, the surface of a hydrophilic solar evaporator is prone to forming a continuous water film, which compromises power generation stability and diminishes or even eliminates the production of streaming potential [16, 17]. Moreover, the precise regulation of nanoconfined channels that govern coupled water‐ion migration remains elusive, and the fundamental mechanisms by which salt‐ion dynamics influence interfacial potential evolution during solar evaporation are still poorly understood [18]. These issues, together with the difficulty of maintaining stable and continuous electric output under fluctuating evaporation conditions, underscore the need for a rationally engineered architecture that simultaneously suppresses salt deposition, optimizes ion transport pathways, and maintains a stable interfacial potential for continuous electricity generation [19].

Recent studies have highlighted the importance of interfacial engineering for regulating water and ion‐selective transport [20]. Core–shell fibrous structures with asymmetric wettability have been used to improve water management and salt resistance in solar evaporation systems [21]. Meanwhile, hydrogel‐electrode interfaces have been shown to enhance ionic transport and interfacial charge transfer for hydrovoltaic output [22]. Moreover, water‐gradient‐driven charge dynamics have further demonstrated that moisture‐induced ion redistribution and interfacial charge regulation can sustain electricity generation during evaporation [23]. Nevertheless, maintaining a stable dry‐wet interfacial potential while simultaneously integrating efficient evaporation, salt resistance, and electricity generation remains challenging.

Inspired by the transpiration architecture of plant leaves, where hydrophilic xylem enables directional water transport while hydrophobic cuticles regulate evaporation [24], we develop an asymmetric Janus nanofiber membrane that exploits a stable dry‐wet interfacial boundary to enable solar‐evaporation‐driven electricity generation. The membrane spatially integrates a hydrophobic carbon‐nanofiber‐decorated photothermal layer with a hydrophilic polydopamine (PDA)/polyethyleneimine (PEI)‐modified nanofibrous substrate. This engineered asymmetry suppresses the formation of a continuous water film that would otherwise dissipate charge separation, and establishes a persistent interfacial potential gradient, enabling sustained electrical output. The optimized Janus evaporator delivers an evaporation‐enhanced open‐circuit voltage of 0.22 V under 1 sun illumination, representing a 57% increase compared to non‐thermal conditions, and achieves scalable output up to 2.21 V via modular integration. This work establishes a dry‐wet heterostructure‐mediated interfacial energy conversion strategy, integrating desalination, directional water transport, and electricity generation within a single platform. Beyond enabling simultaneous freshwater and energy production, it provides fundamental insights into coupled thermal–ionic processes at confined interfaces for solar‐driven systems.

## 2 Experimental Section

### 2.1 Chemical Reagents

Polyurethane (PU) was supplied by Bayer (Hong Kong, China). Carbon nanofibers (CNFs, density: 1.9 g mL^−1^, diameter × length: 100 nm × 20–200 µm) were purchased from Sigma‐Aldrich (USA). The PDMS precursor and curing agent were obtained from Dow Corning (Midland, MI, USA). Ethanol (99.7%) and tris(hydroxymethyl)aminomethane hydrochloride (Tris, ≥98.0%) were purchased from Sinopharm Chemical Reagent Co., Ltd. (China). Dopamine hydrochloride (DA, 98%) was obtained from Accela ChemBio Co., Ltd. All reagents were used as received without further purification.

### 2.2 Fabrication of Asymmetric Janus Nanofiber Membranes

#### 2.2.1 Preparation of CNFs/PU Nanofiber Membrane

A polyurethane (PU) nanofiber membrane, fabricated via electrospinning (details described in our previous work) [25], was used as the substrate. Carbon nanofibers (CNFs) were first dispersed in an ethanol/water mixed solvent (volume ratio 1:4) by ultrasonication (20 kHz) for 15 min to obtain a homogeneous suspension. Under continuous ultrasonication (20 kHz, 240 W), the PU membrane was immersed in the CNFs dispersion, enabling the decoration of CNFs onto the nanofiber surfaces [26]. The resulting membrane is denoted as CNFs/PU.

#### 2.2.2 Preparation of Hydrophobic and Hydrophilic Nanofiber Membranes

To impart hydrophobicity, the CNFs/PU membrane was immersed in a 1 wt.% PDMS solution (containing curing agent in heptane) for Y hours, followed by curing in a vacuum oven at 80°C [27]. The obtained membrane is denoted as PDMS/CNFs/PU‑Y, where Y represents the immersion time in hours. Unless otherwise specified, all characterizations and tests in this work were performed using samples with Y  =  3, denoted as MCP‑3. For hydrophilic modification, dopamine hydrochloride (0.20 g) and polyethyleneimine (PEI, 0.02 g) were dissolved in Tris‑HCl buffer solution (20 mM, pH 8.5) [28], corresponding to a dopamine‐to‐PEI mass ratio of 10:1. The CNFs/PU membrane was immersed in this solution, and polymerization was carried out at room temperature for X hours. The resulting PEI/PDA/CNFs/PU‑X composite is denoted as EACP‑X, where X represents the polymerization time in hours. After reaction, the membranes were thoroughly rinsed with deionized water and ethanol, and then dried in a vacuum oven at 60°C for 1 h. For comparison, membranes modified solely with PDA under identical conditions (without PEI) were prepared and denoted as ACP‑X, where X is the polymerization time of dopamine.

#### 2.2.3 Assembly of Janus Nanofiber Composite for Solar Evaporator

The Janus evaporator was fabricated by assembling a hydrophilic EACP‐X membrane with a hydrophobic MCP‐3 membrane into a bilayer configuration. A polystyrene (PS) foam substrate was employed as a thermally insulating and mechanically supporting platform. The hydrophilic EACP‐X layer was positioned on the PS foam, with both ends threaded through pre‐cut channels and immersed in the simulated seawater to establish a continuous two‐dimensional water‐supply pathway. Subsequently, the superhydrophobic MCP‐3 membrane was placed in conformal contact on top of the EACP‐X layer, serving as the photothermal evaporation interface. This vertically integrated bilayer structure is denoted as Janus‐X. Unless otherwise specified, EACP‐3 was selected as the optimized hydrophilic layer, and the corresponding Janus‐3 device was used for all subsequent photothermal, evaporation, and electrokinetic measurements.

### 2.3 Characterization

The morphology and microstructure of the nanofiber membranes were examined using a field‐emission scanning electron microscope (SEM, Zeiss Supra55, Germany) after sputter‐coating with gold. Fourier transform infrared (FTIR) spectroscopy was performed on a Cary 610/670 spectrometer (Varian, USA) in the range of 4000–400 cm^−1^. X‐ray photoelectron spectroscopy (XPS, ESCALAB 250Xi, USA) was employed to determine the surface elemental composition and chemical states. Optical absorption from 200 to 2500 nm was measured with a UV–Vis–NIR spectrophotometer (Cary 5000, USA). Raman spectra were acquired using a Renishaw in Via confocal Raman microscope. Surface wettability was evaluated by measuring the water contact angle (WCA) with an optical contact angle goniometer (OCA40, Germany).

### 2.4 Solar Driven Interfacial Evaporation (SDIE) Measurements

In the simulated SDIE experiments, the evaporator was constructed using EACP‐X as the hydrophilic water‐transport layer, MCP‐3 as the hydrophobic photothermal layer, and polystyrene (PS) foam as the thermal insulating substrate, enabling continuous self‐sustained water supply. The assembled device was positioned in a beaker containing simulated seawater and irradiated using a xenon lamp solar simulator (PLS‐SXE300+/UV, Perfect Light, Beijing, China). The mass change during evaporation was continuously monitored using a high‐precision electronic balance (PTX‐FA210, Fujian, China), while the surface temperature was recorded in real time with an infrared thermal imaging camera (TiS10, Shanghai, China). All measurements were performed under ambient conditions of 25–30°C and 45%–55% relative humidity. The evaporation performance was evaluated using the following equation:

(1) $$v=\frac{\Delta m}{s\times t}$$

where ∆m represents the change in the mass of seawater (in kg), s represents the irradiated area (in m^2^, the value used in the evaporation test is 10^−4^ square meters), and t represents the duration of evaporation (in hours). In laboratory and outdoor evaporation tests, sample Janus‐3 was used as the standard for the SDIE evaporation test.

### 2.5 Measurement of Electricity Generation Performance of Janus‐3

For electrical output measurements, the hydrophobic photothermal layer (MCP‐3) and the hydrophilic water‐transport layer (EACP‐3) of the Janus‐3 device were independently connected to copper wires using conductive adhesive tape. The photothermal layer served as the working electrode, while the hydrophilic layer acted as the counter electrode. The electrodes were connected to an electrometer (Keithley 6517B) to continuously record the open‐circuit voltage (V_OC_) and short‐circuit current (I_SC_). Electrical signals were acquired at 5 s intervals to capture the dynamic response during evaporation. All measurements were performed under ambient conditions consistent with the solar‐driven interfacial evaporation experiments (25–30°C, 45%–55% relative humidity), ensuring reliable correlation between evaporation behavior and electrokinetic output.

### 2.6 Additional Characterizations and Performance Tests

The wettability of the nanofiber membranes was evaluated by static water contact angle measurements using an optical contact angle goniometer. Photothermal performance was assessed under a xenon lamp solar simulator with tunable irradiance, and the corresponding surface temperature evolution was monitored in real time using an infrared thermal imaging camera. Salt resistance was systematically investigated using NaCl solutions with varying salinities to simulate different seawater conditions. To further evaluate the applicability in complex wastewater treatment, anisole‐in‐water emulsion evaporation experiments were performed to examine the separation and purification capability of the system. Detailed experimental procedures and characterization methods are provided in the Supporting Information.

## 3 Results and Discussion

### 3.1 Preparation and Microstructure of the Janus Nanofiber Composite Membranes

Figure 1 shows the bioinspired design and structural configuration of the Janus evaporator for integrated solar evaporation and electricity generation. Figure 1a schematically illustrates the fabrication of the Janus membrane, inspired by the transpiration mechanism of natural leaves, where the hydrophobic cuticle regulates evaporative water loss and the hydrophilic xylem network sustains continuous capillary‐driven ascent. This functional division between water transport and evaporation control provides the conceptual foundation for constructing an artificial dry‐wet interface that sustains both efficient water supply and stable charge separation. Figure 1b demonstrates the structural design of the leaf‐inspired Janus nanofiber membrane replicating this hydrophilic–hydrophobic dichotomy. The membrane is fabricated on an electrospun polyurethane (PU) nanofiber substrate chosen for its mechanical flexibility and interconnected porous architecture [29, 30, 31]. The top photothermal layer consists of carbon nanofibers (CNFs) decorated onto PU nanofibers followed by a conformal polydimethylsiloxane (PDMS) coating, forming a hydrophobic, light‐absorbing network with high solar absorptance and open pores for vapor escape (Figures S1 and S2). The bottom hydrophilic layer is obtained via in situ polymerization of dopamine and subsequent grafting of polyethyleneimine (PEI), which introduces abundant amino groups and substantially enhances surface hydrophilicity and capillary water transport. This asymmetric configuration establishes a strong unidirectional capillary pressure gradient and a spatially confined dry‐wet interface, a prerequisite for coupling solar evaporation with sustained interfacial electricity generation [14]. Upon hydration, the hydrophilic layer undergoes surface ionization, reducing its local surface potential relative to the persistently dry hydrophobic counterpart and thereby generating a built‐in electric field across the heterojunction. Figure 1c illustrates the limitations of conventional single‐layer hydrophilic membranes. During evaporation, capillary‐driven lateral water diffusion rapidly saturates the entire membrane, forming a continuous surface water film. This homogeneous hydration eliminates spatial contrast in surface ionization, leading to charge equilibration and rapid decay of any initially generated potential [32]. Full wetting also exposes the entire evaporation interface to saline water, accelerating salt crystallization and pore blockage, which further degrades both water transport and electrical output. The operating principle of the Janus evaporator is shown in Figure 1d. The asymmetric architecture maintains a sharp and stable dry‐wet boundary, preserving a persistent surface potential difference between the ionized hydrophilic layer and the dry hydrophobic layer [16]. Evaporation drives directional migration of ionization‐generated cations within the hydrophilic layer toward the interface, creating a sustained ionic flux that reinforces charge separation [33]. Simultaneously, the hydrophobic top layer blocks liquid water permeation and suppresses salt deposition at the evaporation front, ensuring a persistently dry surface that functions as a stable electrical reference. The synergy between accelerated evaporation and salt rejection enables continuous and stable interfacial potential without electrical degradation, fundamentally overcoming the limitations of homogeneous systems.

**FIGURE 1:**
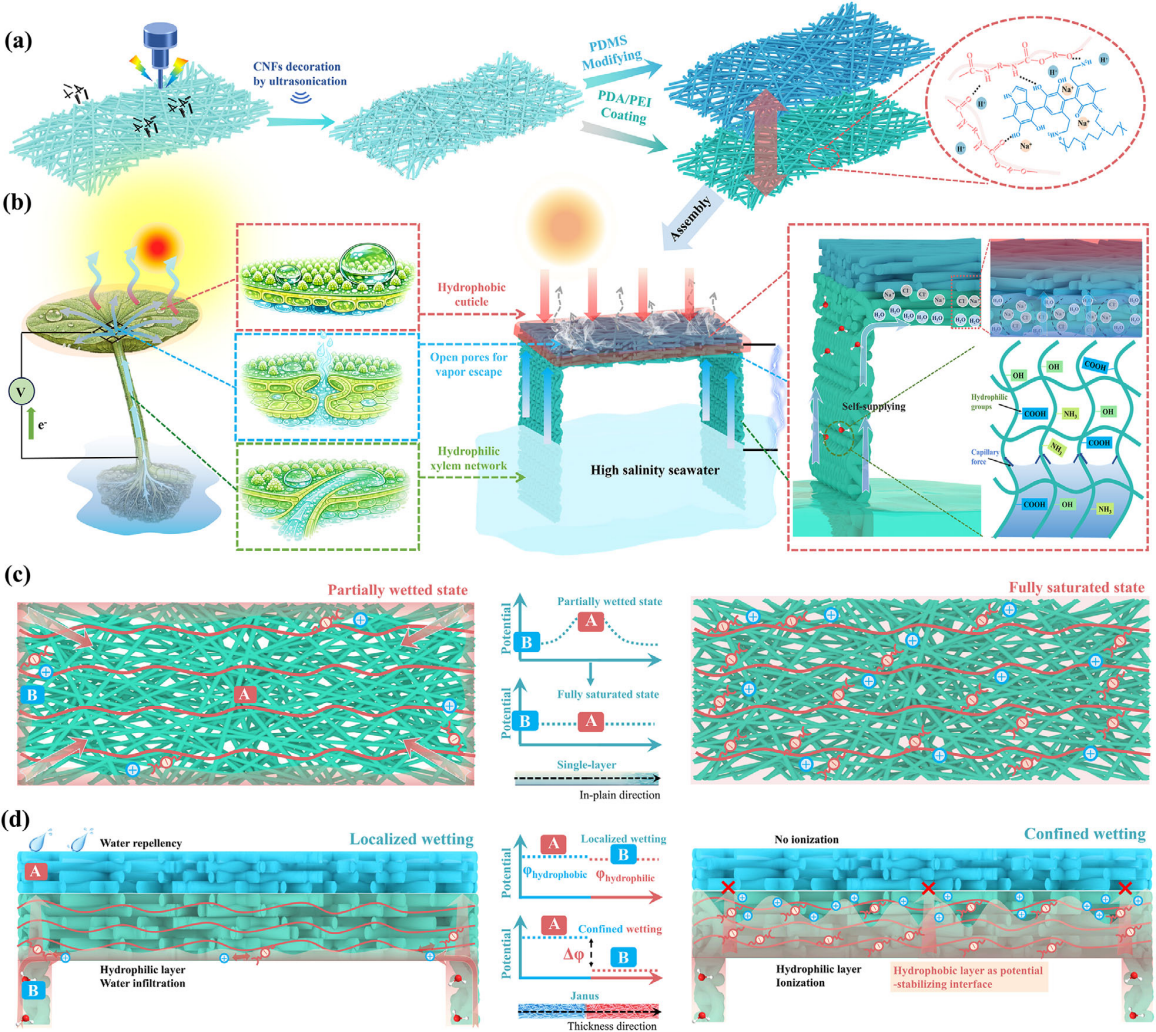
Schematic illustration of a leaf‐inspired Janus evaporator enabling concurrent solar evaporation and electricity generation. (a) Fabrication of the Janus nanofiber membrane, consisting of a hydrophobic nanofiber top layer and a hydrophilic nanofiber bottom layer. (b) Bioinspired design and operating concept of the Janus membrane evaporator for interfacial electricity generation. Comparison of electricity‐generation mechanisms: (c) a conventional single‐layer membrane, where uniform wetting causes lateral ion equilibration and rapid decay of the potential; and (d) the Janus architecture with a confined dry‐wet interface and evaporation‐reinforced ion gradients, maintaining a stable built‐in electric field via asymmetric wetting.

### 3.2 Chemical Composition, Photothermal Conversion Performance and Wettability of the Nanofiber Composites

Figure 2 shows chemical, wetting, and photothermal characterization of the Janus evaporator. FTIR spectra are used to track stepwise surface modification (Figure 2a). Pristine PU nanofibers exhibit characteristic urethane peaks at 3334 cm^−1^ (N–H stretching) and 1726/1597 cm^−1^ (hydrogen‐bonded C═O) [34]. Ultrasonic decoration of CNFs induces a slight blue shift (2–3 cm^−1^) of these bands [35]. Subsequent PDA coating intensifies the N─H stretching region and introduces broad catechol/amine absorption (3300–3400 cm^−1^) [28]. PEI grafting introduces characteristic amine vibrations at 808 cm^−1^, corresponding to aliphatic ─NH_x_ stretching vibrations [36]. Meanwhile, the emergence of a C = N stretching band (1643 cm^−1^) confirms Schiff‐base coupling between PDA quinone groups and PEI amines, indicating successful covalent integration of the hydrophilic coating [37]. For the hydrophobic photothermal layer, PDMS curing yields characteristic Si–C stretching at 798 cm^−1^ and a broad Si─O─Si band (1000–1100 cm^−1^), evidencing crosslinked siloxane network formation. Figure 2b shows a high‐resolution N 1s XPS spectrum of the hydrophilic layer. Deconvolution reveals three nitrogen species attributable to ═N─, ─NH─, and ─NH_2_ groups. The emergence of a C = N component at 401.68 eV verifies Schiff base formation between PDA quinone moieties and PEI amine groups, establishing covalent integration of the hydrophilic coating. The high‐resolution Si 2p XPS spectrum of the hydrophobic photothermal layer (MCP‐3) shows C‐Si and Si‐O‐Si components that confirm PDMS integration while preserving the carbonaceous backbone (Figure 2c). Corresponding survey spectra and additional deconvolution details are provided in Figures S3 and S4.

**FIGURE 2:**
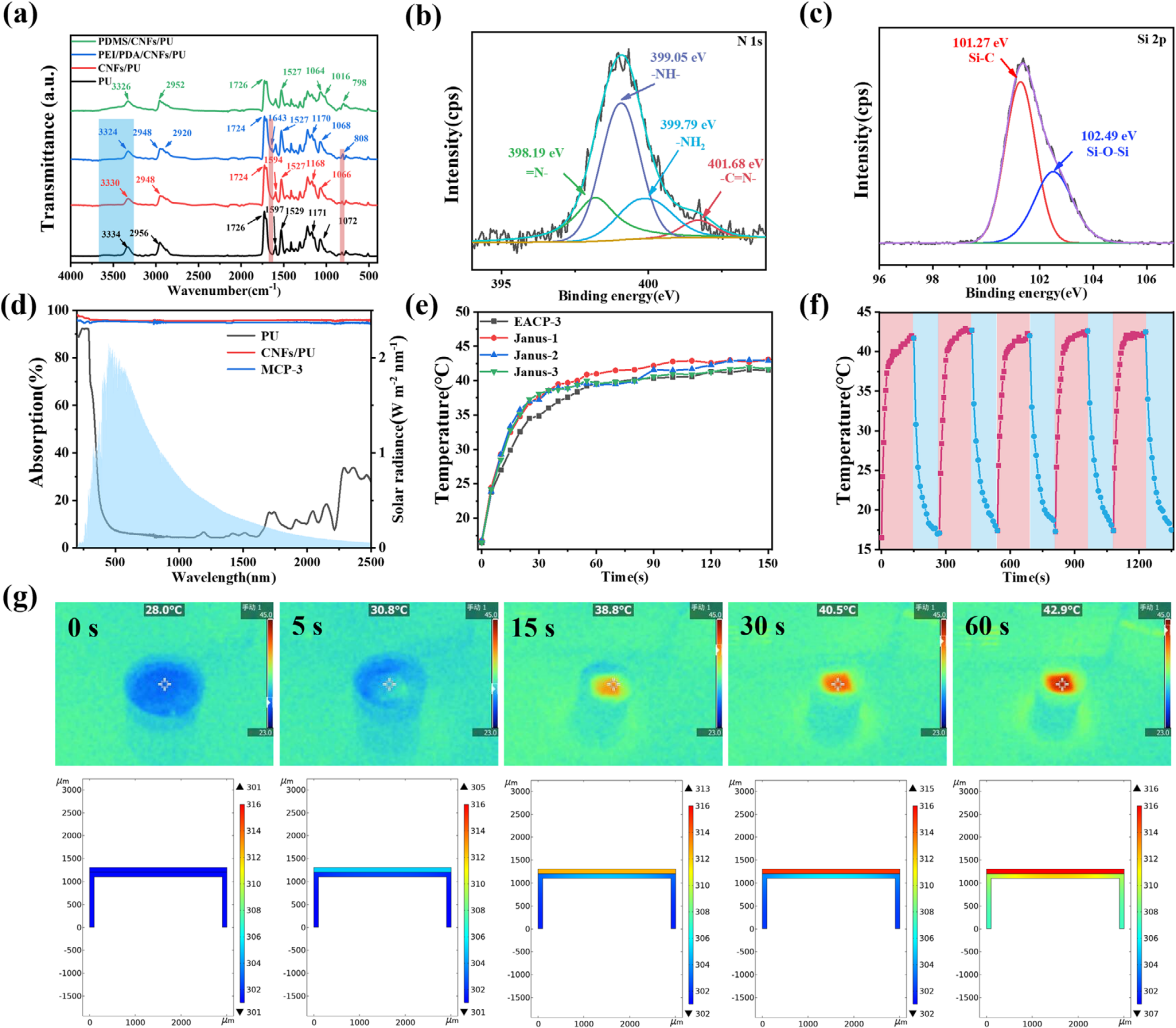
Structure characterization of the Janus nanofiber composites. (a) FTIR spectra of the nanofiber membranes, confirming characteristic functional groups associated with PDA/PEI modification and PDMS coating. (b) High‐resolution N 1s XPS spectrum of EACP‐3, showing fitted peaks corresponding to amine and amide functionalities. (c) High‐resolution Si 2p XPS spectrum of MCP‐3, demonstrating the successful introduction of Si─O─Si and Si─C bonds. (d) UV–Vis–NIR absorption spectra (200–2500 nm), revealing the broadband light‐harvesting capability of CNF‐based photothermal layers. (e) Surface temperature evolution of the different Janus evaporators under 1 sun irradiation, measured in water as a function of illumination time. (f) Heating‐cooling cycling curves of Janus‐3 under repeated light on/off switching, indicating its photothermal stability. (g) Infrared images and corresponding simulated temperature distributions of Janus‐3 at 0, 5, 15, 30, and 60 s of 1 sun illumination. In the schematic, the upper rectangle represents the hydrophobic photothermal layer, while the lower layer with its two bent, downward‐extended ends immersed in water corresponds to the hydrophilic fiber membrane that serves as the water‐supply channel.

Figure 2d displays UV–vis–NIR absorption spectra of different nanofiber membranes. CNFs decoration dramatically enhances broadband solar absorption from 15.36% (PU) to 93.81% (CNFs/PU). Although PDMS introduces a thin transparent coating, MCP‑3 maintains high absorptance (89.21%), attributable to the strong light absorption of CNFs and multiple scattering within the fibrous architecture (Figure S5) [38]. Under 1 sun irradiation. Janus‑1, Janus‑2, and Janus‑3 reach steady‑state temperatures of 43.1, 42.8, and 41.9°C, respectively, within 120 s (Figure 2e), demonstrating rapid photothermal response. Cyclic heating‐cooling performance of Janus‑3 under repeated 1 sun on/off cycles shows nearly overlapping curves (Figure 2f) that indicate excellent photothermal stability and interfacial durability. COMSOL simulations reveal the temperature distribution within Janus‑3 under 1 sun illumination (Figure 2g). The asymmetric architecture spatially segregates the water transport pathway from the photothermal interface, directing heat localization to the confined dry‑wet boundary, fundamentally improving thermal management [9]. Compared to the symmetric hydrophilic membrane (EACP‑3, Figure S6), Janus‑3 exhibits faster heat accumulation and higher steady‑state temperature, confirming that restricted heat dissipation to a confined interfacial zone maintains a sharp temperature gradient essential for thermal‑ionic coupling.

Supplementary characterization of wetting behavior and hydrophilic layer optimization. Figure S7 presents water absorption dynamics of membranes with varying PDA/PEI polymerization times. Pristine PU and CNFs/PU exhibit intrinsic hydrophobicity. PDA deposition alone (ACP‐3) enables complete droplet absorption within 9.1 ± 0.5 s. Subsequent PEI grafting progressively enhances hydrophilicity: EACP‑2 requires 20.3 ± 1.2 s for complete absorption, while extending polymerization to 4 h (EACP‑4) reduces absorption time to 3.5 ± 0.6 s, reflecting increased amine density and improved hydration layer formation. Figure S8 shows water contact angles of the hydrophobic photothermal layer. MCP‑3 exhibits a contact angle of 150 ± 1.6°, confirming superhydrophobicity essential for preventing continuous water film formation and inhibiting salt crystallization at the evaporation interface. Figure S9 compares the absorptance of photothermal layers with varying PDMS curing times; all maintain >89% absorptance, confirming that the conformal PDMS coating does not compromise optical performance.

Together, these results establish that the rationally designed Janus architecture combines rapid water transport, efficient photothermal conversion, and a stable dry‐wet interface. The amine‑rich hydrophilic layer facilitates rapid water and ion transport, while the siloxane‑based hydrophobic layer stabilizes the dry‐wet interface and preserves localized photothermal evaporation. This rational chemical design underpins the membrane's enhanced wettability contrast and salt resistance, sustaining the interfacial ion gradients essential for dynamically reinforcing the built‑in heterostructure potential and achieving stable electricity generation.

### 3.3 Solar‐Thermal Evaporation Performance and Multi‐Environment Operational Stability

Figure 3 shows the solar‐driven interfacial evaporation performance of the Janus evaporator. Figure 3a schematically illustrates the configuration of the SDIE device. A polystyrene (PS) foam serves as the insulating support, featuring two pre‐cut channels (2 cm in length) through which the hydrophilic EACP‑3 membrane is threaded. The EACP‑3 strip extends on both ends into the underlying brine, establishing a continuous two‐dimensional water supply pathway. The superhydrophobic MCP‑3 layer is placed in intimate contact on top of the EACP‑3, functioning as the photothermal absorber. This asymmetric architecture spatially decouples water transport (hydrophilic layer) from photothermal conversion (hydrophobic layer), enabling sustained capillary feeding while maintaining a persistently dry evaporation interface. The hierarchically porous and hollow fiber structure of the composite membrane provides a large specific surface area and interconnected water channels, facilitating rapid water transport and continuous interfacial evaporation. Simultaneously, this architecture stabilizes the ion concentration gradients that dynamically reinforce the interfacial potential, contributing to persistent electrical output. The control experiment yields a baseline seawater evaporation rate of 0.310 kg m^−2^ h^−1^, while the Janus‑2 evaporator achieves a markedly enhanced rate of 1.335 kg m^−2^ h^−1^, representing a 4.3‑fold increase (Figure 3b). Corresponding time‑dependent mass change curves are provided in Figure S10. The solar‐to‐vapor conversion efficiency (η) is calculated as follows: [35]

(2) $$\eta =\frac{m_{eva}}{q_{solar}}\left(\Delta H+C\Delta T\right)$$

m_eva_ represents the evaporation rate under one sun illumination, q_solar_ denotes the intensity of solar radiation, ΔH indicates the change in enthalpy between the liquid and vapor phases under ideal conditions, C refers to the specific heat capacity of seawater (4.2 J g^−1^ K^−1^), and ΔT represents the difference between the evaporation temperature and the initial temperature. As the PDA polymerization time increases, the evaporation rate of the asymmetric Janus evaporator exhibits a corresponding enhancement. The optimized Janus‑3 and Janus‑4 achieve evaporation rates of 1.452 and 1.483 kg m^−2^ h^−1^, respectively, under 1‑sun illumination. Correspondingly, the solar‑to‑vapor conversion efficiencies (η), calculated according to Equation (2), are 83.81%, 91.15%, and 93.09% for Janus‑2, Janus‑3, and Janus‑4, respectively (Figure 3b). To further validate the efficiency calculation, we quantitatively analyzed the heat losses from the Janus‑3 evaporator. Heat loss analysis reveals that the conductive, convective, and radiative heat losses are determined to be 1.98%, 3.80%, and 4.57%, respectively, yielding a total heat loss of approximately 10.35% (see Supporting Information Note S1 for detailed calculations). This value closely aligns with the 8.85% energy loss implied by the 91.15% evaporation efficiency, confirming the consistency and reliability of the efficiency assessment.

**FIGURE 3:**
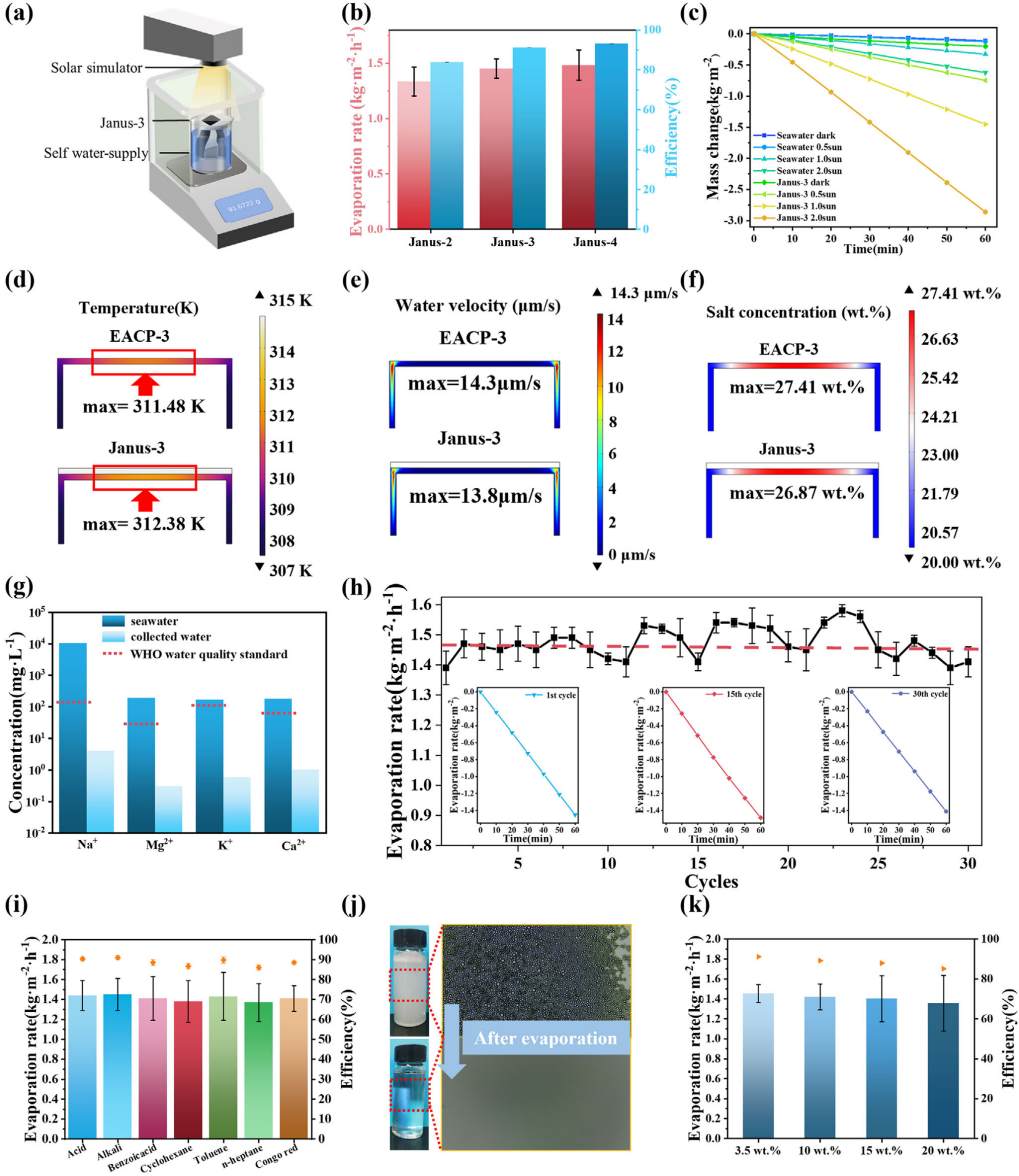
Solar‐driven interfacial evaporation performance of the Janus nanofiber composite evaporator. (a) Schematic illustration of the solar steam‐generation setup. (b) Evaporation rate and corresponding solar‐to‐vapor conversion efficiency. (c) Mass change of seawater over time under 0.5, 1, and 2 sun illumination, with and without the Janus‐3 evaporator. (d) Simulated temperature fields and (e) water‐velocity distributions within the internal channels of EACP‐3 and Janus‐3 during evaporation. (f) Simulated salt concentration distribution in EACP‐3 and Janus‐3 in 20 wt.% simulated seawater under continuous 1 sun illumination for 3 h (two downward‐extending sections in direct contact with water). (g) Concentration variations of four major ions (Na^+^, Mg^2+^, K^+^, Ca^2+^) in seawater before and after desalination. (h) Cycling stability test over 30 evaporation cycles (with representative weight‐loss curves for the first, 15th, and 30th cycles). (i) Evaporation performance of the Janus evaporator in various oil‐in‐water emulsions and under complex environmental conditions. (j) Optical microscopy images and photographs of the anisole‐in‐water emulsion before and after purification. (k) Evaporation rate and efficiency in seawater with salinities of 3.5, 10, 15, and 20 wt.%. All simulations are carried out using COMSOL Multiphysics 5.5.

To further investigate the influence of solar intensity on evaporation performance, both pure seawater and the Janus‐3 evaporator are evaluated under varying illumination conditions (Figure 3c and Figure S11). The corresponding evaporation rate and efficiency are provided in Figure S11. Under dark conditions, both pure seawater and Janus‑3 exhibit minimal evaporation rates of 0.185 and 0.199 kg m^−2^ h^−1^, respectively. As the solar flux increases from 0.5 to 2.0 sun, the evaporation rate of pure seawater progressively rises from 0.219 to 0.622 kg m^−2^ h^−1^, attributed to enhanced molecular kinetics at the liquid–vapor interface under intensified radiative heating. In contrast, Janus‑3 achieves markedly higher evaporation rates of 0.746, 1.452, and 2.861 kg m^−2^ h^−1^ at 0.5, 1.0, and 2.0 sun, respectively, representing 3.4‑, 4.5‑, and 4.6‑fold enhancements over pure seawater under identical illumination. This superior performance originates from the synergistic interplay between the hierarchically porous photothermal layer, which enables multiscale light trapping and efficient solar‑thermal conversion, and the hydrophilic transport layer, which ensures rapid and continuous capillary‑driven water supply to the evaporation interface. The sustained water feeding prevents localized drying and maintains optimal hydration at the evaporation front, thereby maximizing vapor generation under high flux conditions. These results demonstrate that the Janus architecture effectively couples broadband light absorption with unidirectional water transport to achieve exceptional evaporation performance across a wide range of solar intensities.

Figure 3d–f give COMSOL Multiphysics simulations comparing thermal management, hydraulic performance, and salt inhibition between Janus‑3 and single‑layer EACP‑3. The Janus architecture spatially isolates the photothermal interface from the water transport pathway, achieving a more pronounced vertical temperature gradient and enhanced heat localization at the evaporation interface (Figure 3d). Janus‑3 requires a lower transport rate (13.8 µm s^−1^) to sustain continuous water supply compared to single‑layer EACP‑3 (14.3 µm s^−1^), due to the asymmetric capillary pressure gradient created by the distinct surface chemistries of the Janus structure (Figure 3e). Figure S12 shows the simulated negative relative water pressure distribution, confirming continuous upward capillary drive in both systems. In addition, Janus‑3 confines salt primarily within the water‐transport layer, with interfacial concentration stabilizing at 26.87 wt.% (Figure 3f), just slightly above the NaCl saturation point (26.42 wt.%). In contrast, EACP‑3 exhibits salt accumulation up to 27.41 wt.% at the evaporation surface, exceeding the nucleation threshold and leading to crystallization. These results confirm that the hydrophobic photothermal layer not only enhances light absorption but also establishes an asymmetric capillary gradient that stabilizes water and ion transport, enabling sustained desalination while preserving a stable interfacial potential across the dry‐wet heterostructure for continuous electricity generation. Ion concentrations in simulated seawater before and after evaporation are shown in Figure 3g. Post‑evaporation concentrations of Na^+^, Mg^2+^, K^+^, and Ca^2+^ are 4.035, 0.303, 0.591, and 1.021 mg L^−1^, respectively, well within World Health Organization (WHO) drinking water standards. Cycling stability of Janus‑3 is assessed by over 30 repeated evaporation tests (Figure 3h). The evaporation rate remains highly consistent at ∼1.46 kg m^−2^ h^−1^, with mass‑loss curves for the first, 15th, and 30th cycles displaying nearly identical trends, confirming excellent durability.

To assess the practical robustness of the system, the evaporation stability of Janus‐3 is further examined under corrosive and polluted conditions (Figure 3i). The device maintains stable performance due to the robust corrosion resistance of the PEI/PDA‐modified hydrophilic layer and the durable hydrophobicity of the PDMS‐cured photothermal layer. Figure 3j shows optical microscopy images of an anisole‐in‐water emulsion before (up) and after (down) evaporation. The pristine emulsion contains dispersed oil droplets of ∼30 µm diameter, while the condensed water collected after evaporation shows no detectable oil droplets, confirming effective phase separation and water purification. Figure 3k presents evaporation rates of Janus‑3 in simulated brines with increasing salinity (3.5–20 wt.%). The asymmetric wettability confers strong salt resistance through spatial decoupling of the hydrophobic photothermal surface from the hydrophilic water‐transport pathway, preventing surface salt crystallization while maintaining continuous water supply. Even at elevated salinities of 10–20 wt.%, evaporation rates remain relatively stable, decreasing only marginally from 1.421 to 1.356 kg m^−2^ h^−1^. Figure S13 compares the salt resistance of EACP‑3 with a less hydrophilic control (ACP‑3) in 20 wt.% brine. The control exhibits sluggish water transport, leading to salt accumulation and crystallization within 3 h and a progressive decline in evaporation rate. In contrast, Janus‑3 equipped with EACP‑3 maintains a stable evaporation rate of ∼1.35 kg m^−2^ h^−1^ over 3 h, demonstrating that optimized hydrophilicity and rapid water delivery are essential for safeguarding the dry‐wet interface, preventing salt accumulation, and sustaining directional ion transport that preserves the interfacial potential difference. Figures S14 and S15 present outdoor trial results: over 15 consecutive days, the Janus‑3 evaporator produces an average of ∼2.0 kg m^−2^ of water per day, meeting the daily drinking water requirement for a typical adult, with hourly water output showing a clear positive correlation with solar irradiance, demonstrating practical applicability for decentralized water purification.

### 3.4 Water Driven Electricity Generation Performance of Janus‐Based Evaporators

Based on the well‐defined Janus architecture and its interfacial evaporation performance, we next systematically evaluate the electricity‐generation capabilities directly enabled by this asymmetric dry‐wet structure. A series of comparative experiments (Figure 4) are designed to dissect the fundamental contribution of the Janus architecture to power output, establishing direct correlations between structural features and the observed signals. Figure 4a,b show the open‐circuit voltage (V_OC_) and short‐circuit current (I_SC_) of the Janus‐3 evaporator under natural evaporation. V_OC_ rapidly rises to 0.085 V in the initial stage and gradually stabilizes at 0.090 V within 30 min. I_SC_ exhibits a sharp initial increase due to rapid surface charging induced by the wetting of hydrophilic nanofibers, followed by a gradual decrease to 0.40 µA as water infiltration reaches equilibrium and interfacial charge compensation occurs [32]. The sustained V_OC_ and I_SC_ arise from the preserved dry‐wet interface, which maintains a stable potential gradient between the two electrodes. Reversing the measurement probes induces corresponding polarity reversal of V_OC_ and I_SC_ (Figure 4b), confirming that the output originates from a built‐in interfacial electric field with a well‐defined direction. This field arises from the asymmetric electrical double layer structures at the hydrophilic and hydrophobic interfaces, which establish a potential difference determined by their distinct surface potentials [4].

**FIGURE 4:**
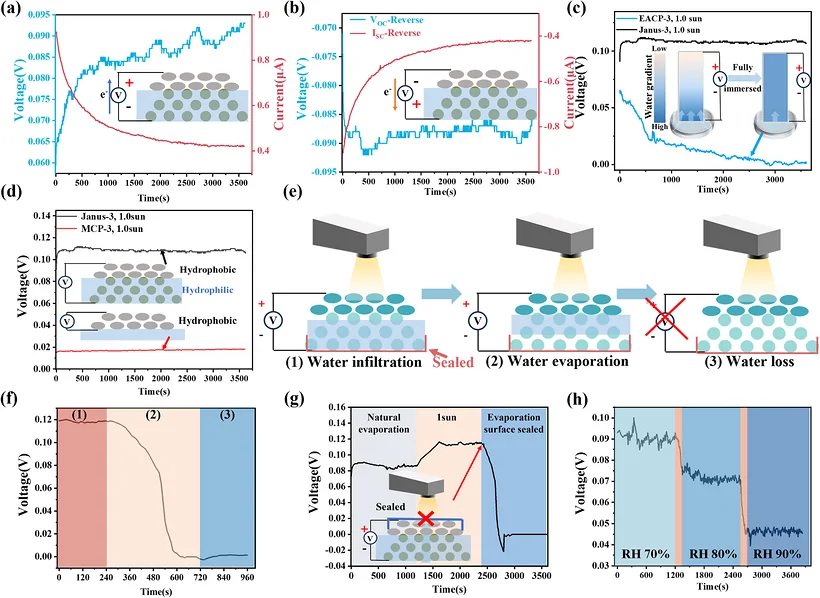
Electricity generation performance of the Janus evaporator in pure water. (a) Open‐circuit voltage (V_OC_) and short‐circuit current (I_SC_) during continuous natural evaporation. (b) V_OC_ and I_SC_ responses upon reversing the electrical measurement probes. Comparison of V_OC_ outputs between the Janus‑3 evaporator and its individual single‑layer components in pure water under 1‑sun illumination: (c) the hydrophilic EACP‑3 membrane and (d) the hydrophobic MCP‑3 membrane. (e) Janus‑3 evaporator under 1‑sun illumination with sealed water‑supply layer, and (f) the corresponding V_OC_ output. (g) Time‐dependent V_OC_ response of Janus‐3 during three sequential stages: natural evaporation, 1‐sun illumination, and sealing of the evaporation surface under continued 1‐sun illumination. (h) V_OC_ response of Janus‐3 under varying ambient relative humidity (RH).

To elucidate the critical role of the asymmetric design, we compare the electricity generation performance of the Janus‑3 evaporator with that of its individual components, i.e., the hydrophilic EACP‑3 layer alone and the hydrophobic MCP‑3 layer alone. When the hydrophilic EACP‑3 layer alone is tested with one end immersed in bulk water and the upper surface initially dry, an initial V_OC_ of ∼0.050 V is observed. As water saturates the nanofiber network over 3000 s, V_OC_ gradually decays to zero (Figure 4c). This result confirms that the built‑in electric field arises from hydration‑induced surface ionization of the hydrophilic layer, which requires a persistently dry hydrophobic counterpart to maintain the potential difference across the interface. The gradual decay of V_OC_ upon complete wetting of the hydrophilic nanofiber membrane directly demonstrates that the interfacial potential originates from asymmetric hydration states. Similarly, when the hydrophobic MCP‑3 layer alone is in direct contact with bulk water, V_OC_ exhibits minor fluctuations and stabilizes at only 0.018 V (Figure 4d). While PDMS modification confers hydrophobicity, prolonged water contact induces partial surface charging without establishing a measurable built‐in field, underscoring the heterostructure (not a single layer) is required to generate a stable interfacial potential.

Figure 4e,f illustrate the operation of Janus‑3 under one‑sun illumination with the water‑transport layer sealed and the evolution of the output voltage. The sealed hydrophilic layer resulted in a finite water supply, and the process proceeds in three stages: (1) initial stage: due to the sealed configuration, only the limited residual water is available to transport to the evaporation interface, sustaining stable V_OC_ output of 0.118 V; (2) intermediate stage: as this finite water is progressively consumed by evaporation, interfacial hydration diminishes, reducing surface ionization and causing V_OC_ to decay; (3) final stage: complete dehydration eliminates the hydration‑induced potential difference, driving V_OC_ to zero over 420 s. Together, these results confirm that sustained hydration is essential for maintaining the built‑in potential, while evaporation serves to reinforce the ionic gradient that amplifies this baseline output [33]. Similarly, under natural evaporation, slow water transport yields a stable Voc of 0.085 V with I_SC_ of ∼0.41 µA (Figure 4g), reflecting the baseline built‑in field established by hydration‑induced surface ionization. Under one‑sun illumination, accelerated evaporation at the dry‐wet interface enhances upward water flux and intensifies the directional ion concentration gradient, amplifying the existing interfacial potential difference and rapidly increasing Voc to 0.115 V (I_SC_ ∼0.65 µA) within 300 s (Figure 4g and Figure S16). To verify the importance of the interfacial evaporation, the evaporation surface is sealed with a transparent plastic film, effectively preventing vapor escape and hence directional water and ion transport. As a result, V_OC_ rapidly decays to near zero. The output voltage is also highly responsive to ambient humidity. As relative humidity at the evaporation surface increases, V_OC_ decreases from 0.090 to 0.045 V (Figure 4h), reflecting diminished steam generation and thus slowed ion transport.

Collectively, these control experiments underscore that the maintenance of a sharp, dynamic dry‐wet interface is indispensable for persistent electricity generation in the Janus system. The rapid decay of electrical output upon interrupted water supply or vapor saturation confirms that sustained voltage generation requires both efficient vapor release and continuous water replenishment, conditions that sustain the ionic gradients responsible for dynamically reinforcing the built‑in interfacial potential.

Based on the experimental and computational evidence, we propose a mechanistic model in which the Janus membrane establishes a dry‐wet heterostructure built‑in field. Kelvin Probe Force Microscopy (KPFM) quantitatively confirms this mechanism, showing a ∼120 mV decrease in the surface potential of the hydrophilic composite (EACP‐3) upon wetting (Figure 5a,b and Figure S17). This potential drop directly evidences the hydration‑induced surface ionization of the hydrophilic layer, which serves as the key driving force for establishing the interfacial potential difference when coupled with the dry hydrophobic counterpart. The surface chemical composition is a decisive factor in tuning this interfacial electrochemistry. Systematic variation of the PDA‐to‐PEI mass ratio reveals a clear structure‑performance relationship: a higher density of oxygen‑containing groups (lower PEI content) favors greater surface potential reduction upon hydration, enhancing the initial output. Conversely, excessive amino group grafting (PDA: PEI = 1: 1) reverses this trend, increasing the surface potential and inverting electrode polarity as the surface becomes dominated by protonatable amines (Figure 5c). This highlights the delicate balance required for optimal performance. Further insight comes from in situ proton tracking using a pH indicator (bromothymol blue). For the PDA‐dominant membrane (PDA: PEI = 1:0), the solution turns yellow within minutes, confirming sustained proton release into the solution, a direct manifestation of the surface dissociation process that establishes the built‑in potential (Figure 5d). In contrast, fabrics with excessive PEI drive the solution alkaline (blue), correlating with their inferior power output. This contrast confirms that the baseline built‑in potential established by controlled surface ionization is essential for the interfacial potential difference.

**FIGURE 5:**
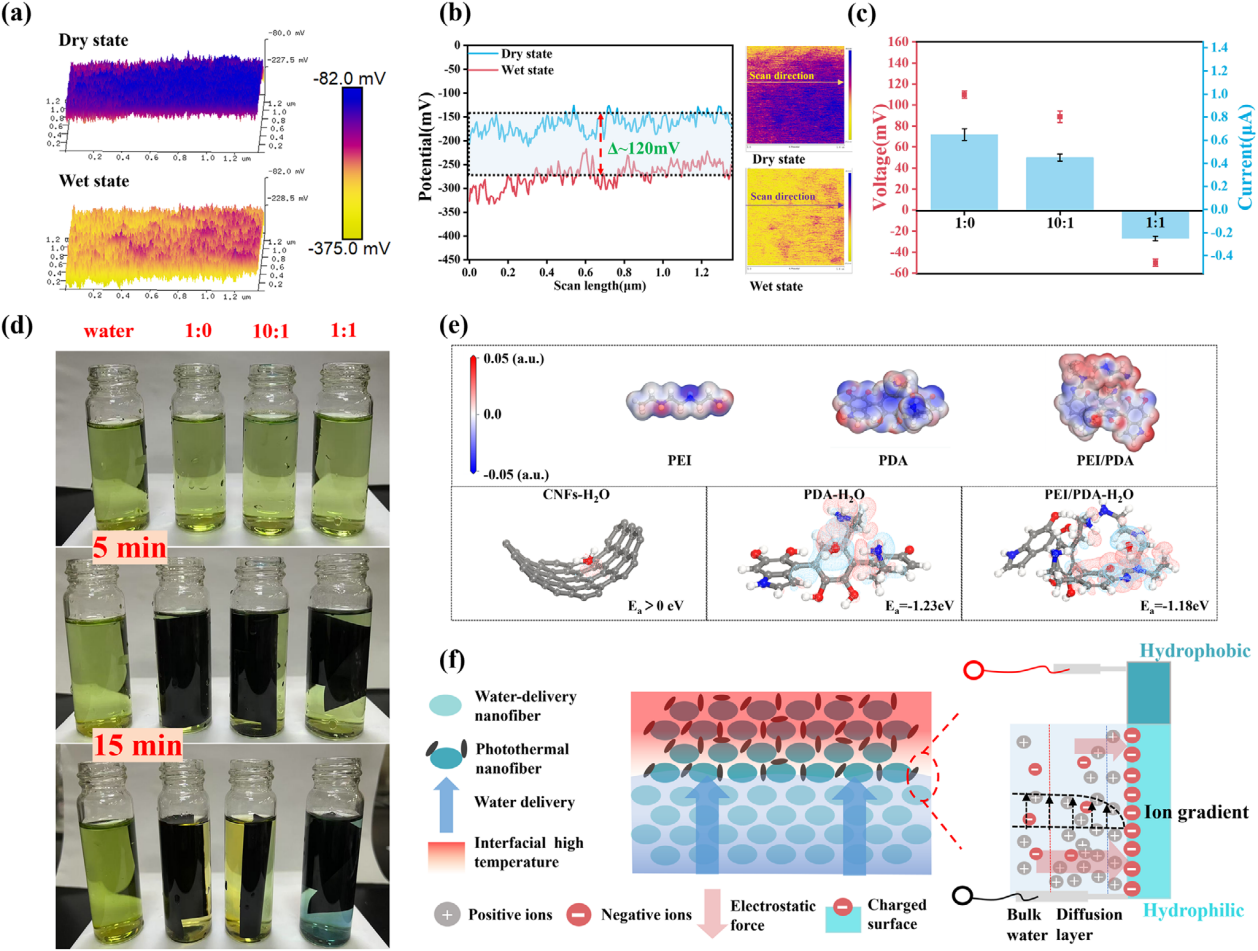
Mechanistic investigation of surface charge generation and interfacial ion transport. (a) Surface potential maps of EACP‐3 before (top) and after (bottom) hydration, highlighting hydration‐induced charge redistribution. (b) Comparison of surface potential distributions of EACP‐3 in the dry and hydrated states. (c) V_OC_ and I_SC_ outputs of the hydrophilic nanofiber membranes prepared with different mass ratios of PDA to PEI. (d) Optical images showing proton generation from various functionalized fabrics immersed in bromothymol blue (BTB) solution, visualizing surface acidification upon hydration. (e) Electrostatic potential maps of different molecular systems and calculated interfacial charge densities of water molecules on CNFs, PDA, and PEI/PDA surfaces. (f) Schematic illustration of electricity generation arising from hydration‐induced surface ionization and evaporation‐driven directional cation migration along the water‐transport pathway, where the EDL provides the local interfacial charge environment and the evaporation‐induced macroscopic concentration gradient drives continuous ion redistribution.

The electrostatic potential (ESP) maps of different molecular systems reveal that the PEI/PDA composite exhibits a broader ESP distribution than PDA alone, with more pronounced positive (red) regions (Figure 5e) [39], indicating enhanced surface charge heterogeneity. This modified charge landscape alters the interfacial interactions with water molecules and may slightly reduce localized adsorption affinity [40, 41]. Density functional theory (DFT) calculations provide atomic‐level validation of these observations. The charge density difference analysis shows that the PDA surface possesses a stronger water adsorption energy (−1.23 eV) compared to the PEI/PDA composite (−1.18 eV) [40], indicating a slight reduction in water adsorption strength upon PEI incorporation. Importantly, this does not contradict the enhanced wettability observed experimentally, as macroscopic wettability depends on collective hydrogen‐bonding interactions rather than single‐molecule adsorption [42, 43]. These results suggest a trade‐off between interfacial charge characteristics and water adsorption behavior. Consistent with experimental observations, although the introduction of PEI somewhat diminishes the surface potential reduction upon hydration, it effectively mitigates interfacial salt crystallization, maintains dry‐wet interface stability by accelerating water transport, and finally optimizes the overall performance of the hydrophilic layer, enabling both sustained water supply and stable power generation.

The electricity generation mechanism of our Janus evaporator operates through a synergistic cascade of coupled physicochemical processes (Figure 5f). First, the engineered Janus architecture establishes a well‐defined interfacial boundary between the hydrophilic transport layer and the hydrophobic confining layer. This asymmetric wettability creates a spatial template for charge separation by confining the electrolyte to the charged transport channels while maintaining a dry counter‐electrode domain. Second, upon wetting, the PDA/PEI‐functionalized surface undergoes hydration‐induced surface ionization, forming a charged solid–liquid interface and the corresponding electric double layer. This surface ionization creates a potential gradient across the Janus interface, with the hydrophilic layer establishing a lower electrochemical potential relative to the dry hydrophobic side. Third, solar‐driven interfacial evaporation induces continuous capillary water flow through the hydrophilic nanofibrous network, generating a macroscopic ion concentration gradient along the water‐transport pathway. This gradient is distinct from the radial ion distribution within the local EDL. The EDL provides the local charge‐selective interfacial environment where mobile ions are enriched near the functionalized surface, whereas the evaporation‐induced concentration gradient drives sustained directional ion redistribution along the transport pathway. This directional ion transport reinforces the pre‐established built‐in heterostructure potential rather than serving as the sole origin of the voltage. Fourth, the optimized PDA/PEI composition provides dual functionality: the balanced polyelectrolyte coating maximizes surface charge density for efficient EDL formation while maintaining the hydrophilic‐hydrophobic balance essential for continuous wicking and salt rejection. This integrated mechanism combining hydration‑induced surface ionization that establishes a built‑in heterostructure potential with evaporation‑driven ion gradients that dynamically amplify this potential enables sustained electricity output without external bias.

Figure 6 presents salinity‐ and illumination‐enhanced electricity generation via ion‐amplified interfacial potential. To distinguish the thermal contribution from the ionic contribution, we first evaluated Janus‐3 in pure water under different illumination conditions. In the absence of added salt ions, the Voc increases from 0.085 V under dark conditions to 0.095 V at 0.5 sun and 0.105 V at 1.0 sun (Figure S18a). Correspondingly, the steady‐state surface temperature increased from approximately 25.3°C in the dark to 38.5°C at 0.5 sun and 42.6°C at 1.0 sun (Figure S18b). These results confirm that increasing thermal input enhances the electrical output by accelerating interfacial evaporation and the associated water and ion transport. Using the solar‐driven evaporation configuration illustrated in Figure S19, we then examine the ionic contribution by varying NaCl concentration under natural evaporation and 1.0 sun illumination (Figure 6a,b). The V_OC_ output increases markedly with salinity, rising from 0.090 V in pure water to 0.280 V at 7.5 wt.% NaCl under illumination. This enhancement does not arise from a distinct voltage‑generation mechanism, but from the cooperative participation of Na^+^ ions with hydration‑generated protons within the electric double layer [44]. Under evaporation, both cationic species are directionally driven along the interfacial concentration gradient; the increased population of mobile Na^+^ in the diffusion layer amplifies charge transport, thereby boosting the electrical output [45]. Solar illumination further amplifies this effect: accelerated evaporation sustains steeper ion concentration gradients, resulting in consistently higher output under illumination than under dark across all tested salinities (Figure 6b) [46]. Figure 6c shows V_OC_ of Janus‑3 in 3.5 wt.% seawater under varying solar intensities (0–2.0 sun). After a brief stabilization period (∼300 s), V_OC_ reaches a steady value that increases monotonically with irradiance. Increasing salinity and increasing light intensity are functionally analogous: both strategies elevate the concentration and mobility of ions, particularly Na^+^ in the diffusion layer, thereby reinforcing interfacial charge transport and enhancing the evaporation‑induced electrical output [47]. Overall, these results show that thermal input and ionic strength can each enhance the voltage output, while their simultaneous action under solar‐driven saline evaporation gives rise to the thermal‐ionic coupling effect. DFT calculations are performed to evaluate the adsorption energies of water molecules and NaCl on the PEI/PDA surface. NaCl exhibits a stronger adsorption energy (−1.35 eV) than water (−1.18 eV) (Figure 6d), indicating stronger ion‐surface affinity. This enhanced interaction favors the accumulation of mobile cations (e.g., Na^+^) near the interface [48]. Consequently, the increased presence of Na^+^ near the interface contributes to a higher density of mobile cations in the diffuse layer, supplying more charge carriers for evaporation‐driven transport and thereby amplifying the EDL‐mediated interfacial potential. Because direct experimental visualization of salt distribution within the porous membrane during evaporation is challenging, COMSOL Multiphysics simulations are employed to map the dynamic salt distribution. Figure 6e presents the simulated salt concentration within the hydrophilic layer after 1 h of evaporation in 3.5 wt.% seawater under 2‐sun illumination, while complementary simulations under lower solar intensities (0–1 sun) are provided in Figure S20. The 2‐sun condition represents an intensified evaporation scenario to highlight ion accumulation behavior. Higher solar intensity progressively increases ion accumulation at the evaporation interface, supplying more mobile Na^+^ to the diffusion layer and strengthening the ion gradient that amplifies the built‑in heterostructure potential. Figure 6f shows continuous 5 h evolution of V_OC_ and I_SC_ under 1 sun illumination in 3.5 wt.% seawater. V_OC_ gradually increases from 0.220 to 0.281 V over the test period. Time‑dependent COMSOL simulation provides salt concentration evolution during the same 5 h evaporation (Figure 6g). The interfacial salt concentration rises from 3.50 to 10.52 wt.%, directly correlating with the observed voltage increase. To validate this mechanism, the simulated interfacial salt concentrations during long‐term evaporation are compared with those obtained under higher‐salinity conditions. Notably, the simulated salt concentrations after 3 h and 5 h evaporation in 3.5 wt.% seawater closely resemble those obtained after 1 h evaporation in 5 wt.% and 7.5 wt.% brines, respectively (Figure S21). Consistently, the experimentally measured V_OC_ values at 3 h (0.245 V) and 5 h (0.280 V) in 3.5 wt.% seawater are nearly identical to those recorded after 1 h evaporation in 5 wt.% and 7.5 wt.% solutions. This agreement between simulation and experiment confirms that progressive interfacial ion enrichment during evaporation directly strengthens the EDL‐mediated potential, thereby enhancing the electrical output. The quantitative series‐connection experiment is performed using one, two, and three Janus‐3 units, showing an increase in output voltage with the number of devices connected in series (Figure 6h). Representative photographs further demonstrate the feasibility of larger arrays: a six‐unit device produced an open‐circuit voltage of approximately 1.64 V, while a nine‐unit device produced approximately 2.21 V under 1 sun illumination (Figure S22 and Figure 6i). The ion‐dependent voltage arises from the coupled effects of cation valence and hydration structure within the electrical double layer (EDL) [16]. Cations with higher valence and larger hydration shells regulate the EDL configuration, thereby modulating the interfacial potential drop [49]. As shown in Figure S23, the measured voltage follows the order CoCl_2_> CaCl_2_> LiCl > NaCl, consistent with ion‐specific effects in classical EDL theory. In particular, cations with larger hydration shells (e.g., Co^2+^) effectively increase the Stern layer thickness, which amplifies the interfacial potential drop and leads to higher voltage output. These results indicate that cation species govern the EDL structure and thus determine the magnitude of the interfacial potential. Extending this mechanism, increasing ion concentration within the same cation system enriches the diffuse layer with mobile charge carriers, which enhances the evaporation‐driven concentration gradient and further amplifies the built‐in potential across the heterostructure [45]. Figure S24 demonstrates long‑term stability: under natural evaporation in 3.5 wt.% simulated seawater, Janus‑3 maintains stable output (V_OC_ ∼0.142 V, I_SC_ ∼0.52 µA) over 30 consecutive 6‑h cycles, confirming excellent durability for extended operation.

**FIGURE 6:**
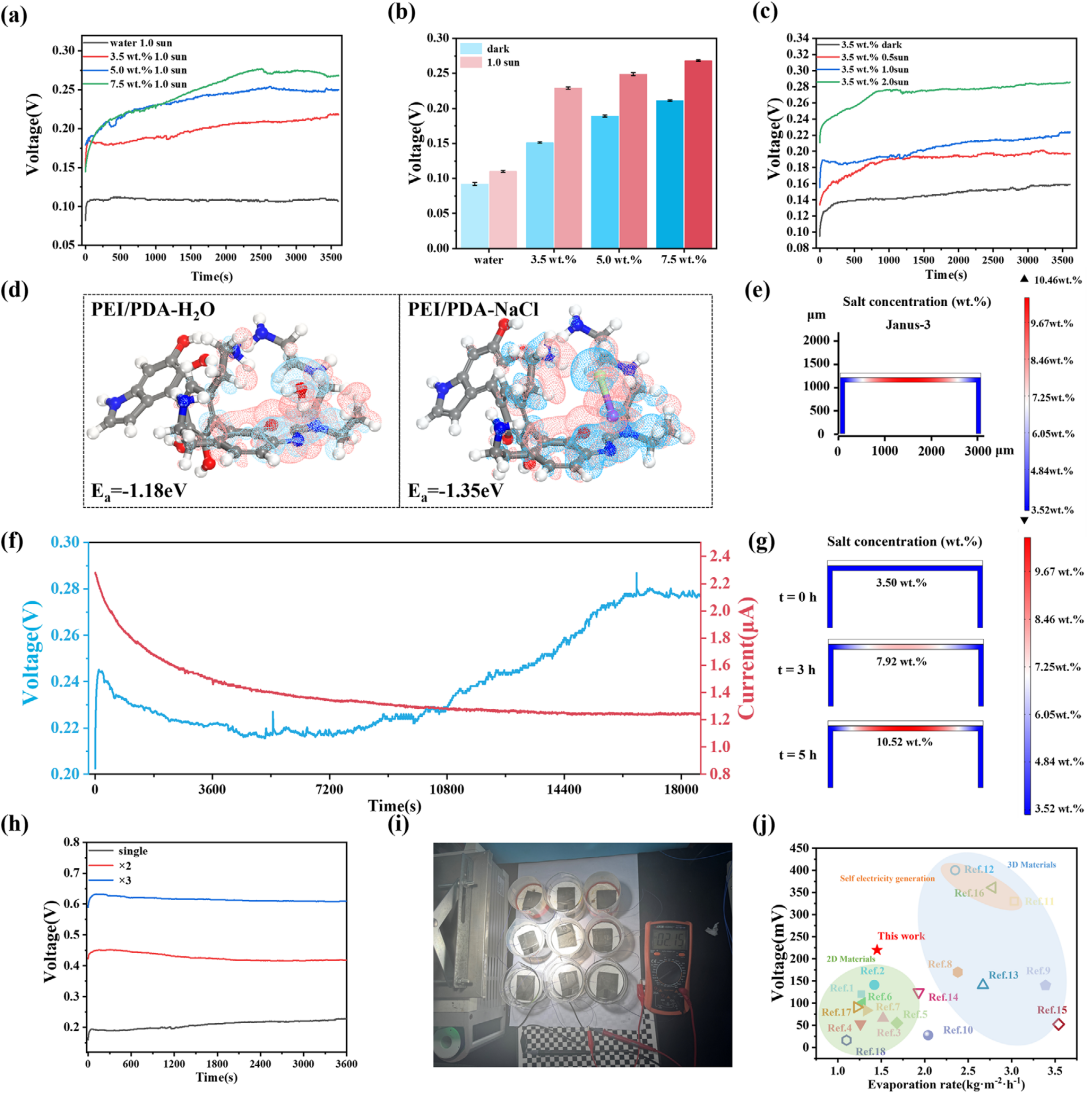
Transition to saline environments: amplification of electrical output by ionic gradients. (a, b) V_OC_ of Janus‑3 in simulated seawater across varying salinities (0–7.5 wt.%) under dark and 1‑sun illumination. (c) V_OC_ of Janus‐3 in 3.5 wt.% simulated seawater under varying solar intensities (0–2.0 sun). (d) Calculated adsorption energies of water molecules and NaCl on the PEI/PDA surface. (e) Simulated salt concentration distribution within Janus‐3 after 1 h of evaporation under 2‑sun illumination (initial salinity: 3.5 wt.%). (f) Continuous 5‑h evolution of V_OC_ and I_SC_ under 1‑sun illumination (initial salinity: 3.5 wt.%). (g) Time‑dependent simulation of salt concentration distribution during interfacial evaporation in 3.5 wt.% seawater under 1‑sun illumination. (h) Enhancement of V_OC_ by connecting multiple Janus‑3 units in series. (i) Photograph of an assembled array of nine devices producing enhanced voltage output under 1‑sun illumination. (j) Comparison of evaporation rates and electrical outputs of Janus‐based evaporators with previously reported systems.

## 4 Conclusions

In summary, we present a bioinspired asymmetric Janus nanofiber membrane that enables simultaneous solar‐driven desalination and electricity generation via a rationally engineered dry‐wet interfacial heterostructure. By spatially integrating a hydrophobic photothermal layer with a hydrophilic water/ion‐transport layer, the system achieves decoupled yet cooperative control over evaporation and fluid transport, effectively suppressing salt accumulation while maintaining a stable interfacial chemical potential gradient. Under 1 sun illumination, the Janus evaporator delivers a voltage output of 0.22 V, 57% higher than that under natural evaporation, and exhibits modular scalability up to 2.21 V with nine units in series. Mechanistic investigations combining experiments, COMSOL simulations, and density functional theory calculations reveal that electricity generation originates from hydration‐induced surface ionization that establishes an intrinsic interfacial potential difference, which is further amplified by evaporation‐driven ion flux within the electric double layer. This work establishes a generalizable strategy for stabilizing interfacial potentials through dry‐wet interface engineering, providing a scalable platform for coupled solar‐thermal‐electric energy conversion. Beyond practical freshwater‐energy co‐generation, it advances the fundamental understanding of thermal‐ionic coupling and interfacial transport in confined systems.

## Author Contributions

**Tingting Zheng**: investigation, formal analysis. **Jun Yan**: writing – review and editing, supervision. **Qin Su**: writing – original draft, writing – review and editing, investigation, visualization, software, data curation. **Haidi Wu**: investigation, conceptualization, formal analysis. **Longcheng Tang**: investigation, resources. **Huaiguo Xue**: investigation, conceptualization, project administration, supervision. **Yongqian Shi**: investigation, resources. **Jiefeng Gao**: writing – review and editing, supervision, funding acquisition, investigation. **Wancheng Gu**: investigation, visualization, project administration. **Xuejun Lai**: investigation, resources. **Di He**: software, investigation, validation.

## Conflicts of Interest

The authors declare no conflicts of interest

## Supporting information




**Supporting File**: advs77250‐sup‐0001‐SuppMat.docx.

## Data Availability

The data that support the findings of this study are available from the corresponding author upon reasonable request.
