# Supplementary material for: Thermal‐Ionic Coupling in Transpiration‐Inspired Janus Nanofiber Membranes: Synergistic Dry‐Wet Interface Engineering for Solar‐Driven Electricity Generation
Source: Adv Sci (Weinh). 2026 Aug 17:e77250. Online ahead of print. doi: 10.1002/advs.77250 (PMC13479851; doi:10.1002/advs.77250)
Supplement: Supplementary file 1 [file ADVS-9999-e77250-s001.docx]

**Supporting information**

**Thermal-Ionic Coupling in Transpiration-Inspired Janus Nanofiber Membranes: Synergistic Dry-Wet Interface Engineering for Solar-Driven Electricity Generation**

Qin Su^a^, Haidi Wu^a^, Tingting Zheng^a^, Di He^a^, Huaiguo Xue*^, a^, Longcheng Tang^b^, Yongqian Shi^c^, Xuejun Lai^d^, Jiefeng Gao*^, a^, Wancheng Gu^a^, Jun Yan*^, a^

^a^ School of Chemistry and Materials, Yangzhou University, No. 180 Road Siwangting, Yangzhou, Jiangsu, 225002, China.

^b^ Key Laboratory of Organosilicon Chemistry and Material Technology of MoE, College of Material, Chemistry and Chemical Engineering, Hangzhou Normal University, Hangzhou, 311121, China.

^c^ College of Environment and Safety Engineering, Fuzhou University, 2 Xueyuan Road, Fuzhou, 350116, China.

^d^ School of Materials Science and Engineering, Key Laboratory of Guangdong Province for High Property and Functional Polymer Materials, South China University of Technology, No. 381, Wushan Road, Tianhe District, Guangzhou 510640, China

* Corresponding authors: chhgxue@yzu.edu.cn; jfgao@yzu.edu.cn;

yan_jun@yzu.edu.cn.

**Contact Angle Measurements**

The wettability of the samples was determined using an optical contact angle measuring system (OCA40, Germany). The spreading and adsorption processes of a 5 μL deionized water droplet deposited on the hydrophilic and hydrophobic composite nanofiber membranes were captured with a high-speed camera. The reported contact angle values represent the average of three measurements taken at different locations on the nanofiber composites surface.

**Photothermal Performance Measurements**

Different samples were placed directly under a xenon lamp at varying solar irradiance levels. The surface temperature of the samples was monitored in real time using an infrared thermal imaging camera (TiS10, Shanghai, China).

**Salt Resistance and Anisole-in-Water Emulsion Evaporation Tests**

To simulate seawater of varying salinity levels, sodium chloride (NaCl) solutions with mass fractions of 3.5wt.%, 10wt.%, 15wt.%, and 20wt.% were employed. Evaporation tests were performed on different samples using the same experimental apparatus immersed in the prepared NaCl solutions.

For the emulsion evaporation experiments, four water-immiscible organic solvents (n-heptane, toluene, cyclohexane, and anisole) were chosen to prepare emulsions containing 2 vol% of the organic phase. The emulsions were formulated by mixing water and the organic solvent at a volume ratio of 50:1, with the surfactant sodium dodecyl sulfonate (SDS) added at a concentration of 0.1 mg/mL. The mixture was then magnetically stirred at 1000 rpm for 24 hours to achieve a stable emulsion. The evaporation testing procedure remained consistent with the salt resistance test described earlier, with the only modification being the replacement of the simulated seawater with the respective prepared emulsion.

Figure S1 TEM of the CNFs.

Figure S2 SEM of the PU (a), the CNFs/PU (b), the ACP-3 (c), the EACP-2 (d), the EACP-3 (e) and the EACP-4 (f).

Figure S3 (a) Comparison of the N contents before and after PEI grafting onto the PDA coating. (b) High-resolution N 1s spectrum and peak fitting of ACP-3. (c, d) high-resolution C 1s spectra of ACP-3 and EACP-3, respectively.

Figure S4 C 1s peak fitting of MCP-3 hydrophobic layer.

Figure S5 The mechanism underlying the efficient light absorption of the Janus evaporator and its salt-resistant performance under high salinity conditions.

Figure S6 (a-c) IR images and (d-f) corresponding simulated temperature distributions of EACP-3 under 1 sun illumination at 15, 30, and 60 s.

Figure S7 Photographs of water droplet diffusing into different hydrophilic nanofibrous composites.

Figure S8 Contact Angles of CNFs/PU composites for different PDMS curing time.

Figure S9 The light absorption spectra of photothermal layer with different PDMS curing time.

Figure S10 Time-dependent mass changes of seawater during evaporation without an evaporator and with the Janus-2, Janus-3, or Janus-4 evaporator under 1-sun illumination.

Figure S11 Evaporation rates and efficiencies of seawater with and without the Janus-3 under 0.5, 1.0, and 2.0 sun illumination.

Figure S12 Simulated water pressure distributions inside (a) EACP-3 and (b) the Janus-3 during evaporation.

Figure S13 (a) Image of salt precipitation on the surface of single layer evaporator after 3 h evaporation based on ACP-3 in 20 wt. % salinity simulated seawater. (b) Evaporation rates of Janus evaporators containing different hydrophilic layers during 3h of continuous operation in 20 wt. % NaCl solution

Figure S14 Daily water output from the Janus-3 evaporation device under natural sunlight over a period of 15 days. (From November 9th to 24th, Yangzhou, China)

Figure S15 Outdoor mass loss of evaporator per hour and its corresponding natural sunlight intensity.

Figure S16 Time-dependent I_SC_ response of Janus-3 during three sequential stages: natural evaporation, 1-sun illumination, and sealing of the evaporation surface under continued 1-sun illumination.

Figure S17 Surface-potential distributions of EACP-3 measured by Kelvin probe force microscopy (KPFM) in the dry and hydrated states.

Figure S18 (a) Open-circuit voltage and (b) surface-temperature evolution of Janus-3 in pure water under dark, 0.5 sun, and 1.0 sun conditions.

Figure S19 Representative photograph of the Janus-3 device operating under coupled thermal-ionic conditions during solar-driven interfacial evaporation in 3.5 wt.% simulated seawater under 1 sun illumination.

Figure S20 Simulated salt concentration distribution in Janus-3 under 0, 0.5 and 1.0 sun illumination for 1 hour (in 3.5 wt. % seawater).

Figure S21 Simulated salt concentration distributions in Janus-3 in (a, b) 5.00 wt. % and (c, d) 7.50 wt. % salinity simulated seawater under 1 sun illumination after one-hour evaporation.

Figure S22 Photograph of six Janus-3 devices connected in series, producing an open-circuit voltage of approximately 1.64 V under 1-sun illumination.

Figure S23 Open-circuit voltage (V_OC_) and short-circuit current (I_SC_) outputs of Janus-3 in solutions containing different cations (Na^+^, Li^+^, Ca^2+^, and Co^2+^).

Figure S24 Cycling stability of Janus-3 in 3.5 wt.% simulated seawater under natural evaporation, showing the open-circuit voltage (V_OC_) and short-circuit current (I_SC_) over 30 consecutive 6-h cycles.

**Note S1. Calculations of energy under 1 sun^[1]^**

Energy input during solar evaporation, Ein = I_in_ ×A×t = 360 J

Where A is the irradiated area of MCP-3 (1×10^-4^ m^2^) and t is the time duration of the solar evaporation (3600 s).

S1.1 Conductive heat loss

The heat dissipated into the water (J_conductive_)can be calculated with the following

formula:

$$\text{J}_{\text{conductive}}\text{=CmΔ}\text{T}_{\text{bulk}}$$

(1)

Where C is the specific heat capacity of water (4.2 kJ °C^-1^ kg^-1^), m represents the weight of bulk water, and ΔT_bulk_ (31.1 – 29.4 °C) represents the increased temperature of the bulk water after stable steam generation.

J_cond_=C·m·ΔT_bulk_= 7.14 J

Conductive heat loss = J_cond_ /E_in_ ×100% =1.98%

S1.2 Convective heat loss

The heat dissipates into the environment (J_convection_) can be calculated with the

following formula:

$$\text{J}_{\text{convection}}\text{=hA(}\text{T}_{\text{MCP}\text{s}}\text{-}\text{T}_{\text{environment}}\text{)}$$

(2)

Where h denotes convection heat transfer coefficient (5 W m^-2^ K^-1^), A is the area of the evaporation surface (1.0×10^-4^ m^2^), T_MCPs_ (313.8 K) and T_environment_ (306.2 K) are the temperatures of MCP-3 and environment.

Convective flux, P_conv_ = J_conv_ /A = h (T_MCPs_ - T_environment_) = 38.00 W m^-2^

Convective heat loss = P_conv_ /I_in_ ×100% = 38.00 W m^-2^/1000 W m^-2^ ×100% =3.80%

S1.3 Radiative heat loss

Heat radiated into the air (J_radiative_) can be calculated with the following formula:

$$\text{J}_{\text{radiative}}\text{=εσA(}\text{T}_{\text{MCP}\text{s}}^{\text{4}}\text{-}\text{T}_{\text{environment}}^{\text{4}}\text{)}$$

(3)

Where 𝜀 denotes the emission of the absorber, 𝜎 is the Stefan-Boltzmann constant (5.67×10^-8^ W m^-2^ K^-4^). A is the area of the evaporation surface (1.0×10^-4^ m^2^), T_MCPs_ (313.8 K) and T_environment_ (306.2 K) are the temperatures of MCP-3 and environment.

Radiation flux, Prad = Φ/A = ε σ (T^4^_MCPs_–T^4^_environment_) = 45.70 W m^-2^

Radiative heat loss = P_rad_ /I_in_ ×100% = 45.70 W m^-2^/1000 W m^-2^ ×100% =4.57 %

**Note S2 Stern model for EDL^[2]^**

The voltage is determined by the following equation

$$\text{Φ=σ}\text{⋅}\text{d/(ε}\text{⋅}\text{ε}_{\text{0}}\text{)}$$

(4)

where σ, d, ε, and ε_0_ represent the surface charge density, the thickness of the Stern layer, the dielectric constant of the liquid at the solid surface, and the vacuum permittivity, respectively.

**Note S3 Theoretical calculation**

All density functional theory (DFT) calculations are carried out using the CASTEP module in Materials Studio 2020. The exchange-correlation energy is described within the generalized gradient approximation using the Perdew-Burke-Ernzerhof (PBE) functional. The kinetic-energy cutoff of 600 eV. Brillouin-zone integrations are performed using Monkhorst-Pack k-point meshes chosen to ensure a k-point spacing finer than 0.03 Å^-1^. All structures are fully relaxed until the total energy changed by less than 1×10^-5^ eV per atom, the maximum force on any atom was below 0.01 eV Å^-1^, and the maximum atomic displacement is less than 5×10^-4^ Å; for surface models, the bottom substrate layers are fixed and a vacuum region of 15 Å is applied along the surface normal.

Binding energies are evaluated as

$$\text{E}_{\text{b}}\text{=}\text{E}_{\text{complex}}\text{-}\text{E}_{\text{substrate}}\text{-}\text{E}_{\text{adsorbate}}$$

(5)

Differential charge density (CDD) plots are obtained from

Δρ = ρ_complex_ – ρ_substrate_ − ρ_adsorbate_.

$$\Delta\rho\text{=}\text{ρ}_{\text{complex}}\text{-}\text{ρ}_{\text{substrate}}\text{-}\text{ρ}_{\text{adsorbate}}$$

(6)

Electrostatic potential (ESP) maps are calculated using the DMol3 module. All structures are first fully optimized under the density functional theory framework employing the generalized gradient PBE exchange-correlation functional. The numerical integration grid is set to fine accuracy. Self-consistent field (SCF) cycles are run until convergence to a total energy of 1×10^-6^ Ha and a charge density of 1×10^-5^ Ha. Geometric optimization thresholds are set to a maximum force of 0.002 Ha Å^-1^ and a maximum atomic displacement of 0.005 Å. Subsequently, ESP is mapped onto the molecular van der Waals surface, yielding the color-coded maps presented in this study.

**Note S4 COMSOL Simulation^[3]^**

The temperature distribution, isothermal profiles, and salt concentration within the Janus-3 are simulated using COMSOL Multiphysics software (version 5.5). A numerical model is developed to analyze the heat and salt concentration distributions in the Janus-3 structure. The Janus-3 model consists of an upper hydrophobic photothermal layer (3000 μm × 100 μm) and a lower hydrophilic layer containing a central suspended region (3000 μm) and two downward-extending sections (~1200 μm each) in direct contact with water. A uniform solar flux of 1 kW·m^-2^ is applied to the upper surface

The simulation incorporates a dilute species transport module coupled with a heat transfer in liquids module.

**S4.1 Heat Distribution Simulation**

A constant solar irradiance of 1 kW·m^-2^ is applied to the top surface to drive the evaporation process. The water influx into the evaporator is balanced by the outgoing vapor flux due to evaporation. Heat losses from the top of the evaporator are accounted for through convection, radiation, and the latent heat carried by vaporization. The simulation is performed by solving the following set of equations:

$$\text{ρ×C}_{\text{p,tot}}\text{u×}\text{∇}\text{T+}\text{∇}\text{×q=Q+}\text{Q}_{\text{eva}}\text{+}\text{Q}_{\text{c}}\text{+}\text{Q}_{\text{e}}$$

(7)

$$\text{Q}_{\text{eva}}\text{=-}\text{h}_{\text{evap}}\text{m}_{\text{eva}}$$

(8)

$$\text{Q}_{\text{c}}\text{=-}\text{h}_{\text{c}}\text{(T-}\text{T}_{\text{e}}\text{)}$$

(9)

$$\text{Q}_{\text{e}}\text{=-}\text{E}_{\text{m}}\text{(}\text{T}^{\text{4}}\text{-}\text{T}_{\text{e}}^{\text{4}}\text{)}$$

(10)

The parameters in the heat-transfer model for the Janus-3 evaporator are defined as follows: 𝜌 is the density; C_p,tot_ is the total heat capacity at constant pressure; q is the conductive heat flux; k_eff_ is the effective thermal conductivity; u is the velocity field; Q_evap_ is the latent heat source, h_evap_ is the vaporization enthalpy, m_eva_ is the evaporation flux on the evaporating surface, Q_c_ is the heat convection on the surface, h_c_ is the convection coefficient (5-10 W m^−2^ K^−1^), Q_e_ is the heat radiation on the surface, Em is the Stefan-Boltzmann constant (5.67 × 10^−8^ W m^-2^ K^−4^), T_e_ is the environment temperature (298.15 K), and Q is the total energy input at the top surface, defined as α q_i_, with q_i_ being the solar intensity (1 kW·m⁻²) and α the solar absorptance of photothermal layer (89%).

**Simulation of salt distribution**

To simulate the salt and heat distribution of Janus-based evaporator, the mass flux of NaCl (J_eva_) and mass loss is applied on the evaporation interface:

$$\text{J}_{\text{eva}}\text{=}\frac{\text{m}_{\text{eva}}\text{∙c}}{\text{ρ}}$$

(11)

$$\text{m}_{\text{eva}}\text{=-}\text{k}_{\text{m}}\frac{\text{M}}{\text{R}}\text{(}\frac{\text{P}_{\text{MCP}}^{\text{v,sat}}}{\text{T}}\text{-}\text{H}_{\text{R}}\frac{\text{P}_{\text{envir}}^{\text{v,sat}}}{\text{T}_{\text{e}}}\text{)}$$

(12)

$$\text{P}_{\text{x}}^{\text{v,sat}}\text{=}\text{P}_{\text{0}}\text{exp(}\frac{\text{h}_{\text{eva}}}{\text{R}}\text{M(}\frac{\text{1}}{\text{T}_{\text{0}}}\text{-}\frac{\text{1}}{\text{T}}\text{))}$$

(13)

Where M represents the molar mass of water, given as 18 × 10^−3^ kg mol^−1^, the universal gas constant is denoted as R, with a value of 8.314 J mol^−1^ K^−1^, The pressures of water vapor at the surface temperature T and the environmental temperature T_e_ are represented as $\text{P}_{\text{MCP}}^{\text{v,sat}}$ and$\text{P}_{\text{envir}}^{\text{v,sat}}$ , respectively. Additionally, H_R_ denotes the relative humidity, while k_m_ signifies the convective mass transfer coefficient. P_0_ is the standard pressure, and T_0_ refers to the boiling point of water.

Table S1. Physical parameters, initial conditions, and boundary conditions used in the COMSOL simulations

| **Parameter** | **Symbol** | **Value** | **Description** |
| --- | --- | --- | --- |
| Initial NaCl concentration | c_0_ | 3305 mol m^-3^ | 20 wt. % NaCl model |
| Molar mass of water | M | 18 ×10^-3^ kg mol^-1^ | Used in the evaporation-flux expression |
| Universal gas constant | R | 8.314 J mol^-1^ K^-1^ | / |
| Convective mass-transfer coefficient | k_m_ | 0.01 m s^-1^ | Vapor transport from the evaporation surface |
| Water activity | a_w_ | 1.0 | Used in the evaporation-flux expression |
| Ambient relative humidity | H_R_ | 40% | / |
| Temperature above evaporation surface | T_a1_ | 298.15 K | Evaporation surface temperature |
| Ambient temperature at side boundaries | T_a2_ | 298.15 K | Ambient temperature on the side |
| Enthalpy of vaporization | h_v_ | 2256 kJ kg^-1^ | / |
| Standard atmospheric pressure | P_0_ | 101325 Pa | / |
| Reference boiling temperature of water | T_0_ | 373.15 K | / |
| Temperature at the upper evaporation-surface boundary | T_1_ | Solved variable (not prescribed) | The boundary probe on the upper surface of the evaporator |
| Evaporation mass flux | m_1_ | ms_ur1_×3600 kg m^-2^ h^-1^ | The boundary probe on the upper surface of the evaporator |
| Evaporation mass flux | m_sur1_ | $\text{-}\text{k}_{\text{m}}\frac{\text{M}}{\text{R}}\text{(}\frac{P_{m1}\cdot\alpha_{w}}{T_{1}}\text{-}\text{H}_{\text{R}}\frac{P_{a1}}{\text{T}_{a1}}\text{)}$ | The evaporation rate on the upper surface |
| Pressure | P_m1_ | $P_{0}exp(h_{v}\frac{M}{R}(\frac{1}{T_{0}}-\frac{1}{T_{a1}}))$ | The water vapor pressure on the upper surface of the material |
| Pressure | P_a1_ | $P_{0}exp(h_{v}\frac{M}{R}(\frac{1}{T_{0}}-\frac{1}{T_{1}}))$ | The water vapor pressure of the air on the upper surface of the evaporator |
| Water-supply/immersed boundaries | Velocity inlet | $U_{n}=\frac{m_{sur1}}{\rho}$ | Inlet velocity is coupled to the evaporation mass flux so that evaporated water is replenished |
| Evaporation boundary | Pressure outlet | Pa gauge pressure | Allows fluid outflow at the evaporation interface |
| All fluid domains | Gravity | $\rho g,g=(0,-g)$ | Includes hydrostatic pressure contribution |
| Inlet | Backflow suppression | Enabled | Prevents numerical reverse flow |
| Outlet | Backflow suppression | Enabled | Stabilizes the pressure outlet |
| Water-supply/immersed boundaries | Initial concentration | c(t=0)=c_0_ mol m^-3^ | Uniform initial salinity |
| Evaporation interface | General inward flux | J_0_=−m_sur1_ c_0_/ρ | Salt redistribution caused by water loss at the evaporating boundary |
| Impermeable outer boundaries | No flux | −n⋅N=0 | Salt cannot cross non-supply/non-evaporation boundaries |
| Convective contribution at evaporation flux boundary | Included | Enabled | Coupled species transport to evaporation-driven liquid motion |

Table S2 Key parameters and performance metrics of representative solar-driven evaporators with simultaneous electricity generation reported in the literature (related to Figure 6j).

| Study in Figure 6j | Light intensity  (W m_-_^2^) | Salinity (wt. %) | The mechanism of power generation |
| --- | --- | --- | --- |
| 1 | 1000 | 3.5 | Thermoelectric module |
| 2 | 1000 | 3.5 | Thermoelectric module |
| 3 | 1000 | 3.5 | Thermoelectric module |
| 4 | 1000 | 3.5 | Thermoelectric module |
| 5 | 1000 | 3.5 | Hydrovoltaic |
| 6 | 803 | 3.5 | Hydrovoltaic |
| 7 | 1000 | 3.5 | Thermoelectric module |
| 8 | 1000 | 3.5 | Thermoelectric module |
| 9 | 1000 | 3.5 | Thermoelectric module |
| 10 | 1000 | 3.5 | Thermoelectric module |
| 11 | 1000 | 3.5 | Thermoelectric module |
| 12 | 1000 | 3.5 | Hydrovoltaic |
| 13 | 1000 | 3.5 | Thermoelectric module |
| 14 | 1000 | 3.5 | Thermoelectric module |
| 15 | 1000 | 3.5 | Hydrovoltaic |
| 16 | 1000 | 3.25 | Thermoelectric supercapacitor |
| 17 | 1000 | 3.25 | Redox couple |
| 18 | 1000 | 3.5 | Thermoelectric supercapacitor |

The numbering in the first column corresponds to the order of the studies displayed in Figure 6j and does not represent the reference numbering of the manuscript. The corresponding references are listed below.

1.Y. Li, C. Fu, Z. Wang, L. Huang, L. Chen, G. Liao, Q. Zheng,Y. Ni, *J. Cleaner Prod.* **2024,** 458, 142416.

2. Y. Xu, Z. Guo, J. Wang, Z. Chen, J. Yin, Z. Zhang, J. Huang, J. Qian,X. Wang, *ACS Appl. Mater. Interfaces* **2021,** 13, 27129-27139.

3.Z. Fu, D. Zhong, S. Zhou, L. Zhang, W. Long, J. Zhang, X. Wang, J. Xu, J. Qin, J. Gong, L. Li, L. Xia, B. Yu,W. Xu, *Adv. Sci.* **2024,** 11, 2406474.

4.A. Ghaffar, M. Usman, M. U. Khan,M. Hassan, *J. Cleaner Prod.* **2024,** 446, 141374.

5.R. Xu, H. Cui, N. Wei, J. Zhao, X. Song, Y. Han, J. Yang, A. Wang,M. Zhao, *Desalination* **2024,** 570, 117094.

6.Y. Duan, M. Weng, W. Zhang, Y. Qian, Z. Luo,L. Chen, *Energy Convers. Manage.* **2021,** 241, 114306.

7.F. L. Meng, M. Gao, T. Ding, G. Yilmaz, W. L. Ong,G. W. Ho, *Adv. Funct. Mater.* **2020,** 30, 2002867.

8.Z. Fan, J. Ren, H. Bai, P. He, L. Hao, N. Liu, B. Chen, R. Niu,J. Gong, *Chem. Eng. J.* **2023,** 451, 138534.

9.L. Chen, J. Ren, J. Gong, J. Qu,R. Niu, *Chem. Eng. J.* **2023,** 454, 140383.

10.W. Lu, D. Jiang, Z. Wang, X. Zhang, Q. Ding, Z. Zhang, X. Liu, L. Bai, Z. Li,Y. Liu, *Chem. Eng. J.* **2024,** 496, 154361.

11.G. Hu, H. Liu, K. Liu, H. Wang, X. Wen, L. Liu, Y. She, L. Feng, R. Niu,J. Gong, *Adv. Funct. Mater.* **2025,** 35, 2423781.

12.Y. Ma, Y. Hu, N. Li, Y. Wang, J. Yu,Z. Hu, *Adv. Funct. Mater.* **2025,** 35, 2422725.

13.R. Niu, J. Ren, J. J. Koh, L. Chen, J. Gong, J. Qu, X. Xu, J. Azadmanjiri,J. Min, *Adv. Energy Mater.* **2023,** 13, 2302451.

14.	X. Jing, L. Chen, Y. Li, H. Yin, J. Chen, M. Su, F. Liu, T. Abdiryim, F. Xu, J. You,X. Liu, *Small* **2024,** 20, 2405587.

15.B. Nie, W. Zhang, X. Dou, Y. Meng, X. Zhao, Y.-C. Wu,H.-J. Li, *J. Mater. Chem. A* **2024,** 12, 293-302.

16.Y. Wan, Y. Hu, H. Tu, W. Zhuang, X. Geng, T. Zhang, J. Zhang, M. Wen,P. Yang, *Small* **2024,** 20, 2302943.

17.B. Gong, H. Yang, S. Wu, Y. Tian, X. Guo, C. Xu, W. Kuang, J. Yan, K. Cen, Z. Bo,K. Ostrikov, *Carbon* **2021,** 171, 359-367.

18.Q. Shen, Z. Ning, B. Fu, S. Ma, Z. Wang, L. Shu, L. Zhang, X. Wang, J. Xu, P. Tao, C. Song, J. Wu, T. Deng,W. Shang, *J. Mater. Chem. A* **2019,** 7, 6514-6521.

**Reference**

[1]	Q. Su, J. Yan, W. Xiao, Y. Liu, L. Li, J. Wang, X. Huang, H. Xue,J. Gao, *Chem. Eng. J.* **2024,** 490, 151814.

[2]	S. Sun, H. Li, M. Zhang, B. Sun, Y. Xie, W. Zhou, P. Yang, H.-Y. Mi, Z. Guo, C. Liu,C. Shen, *Small* **2023,** 19, 2303716.

[3]	J. Yan, T. Cui, Q. Su, H. Wu, W. Xiao, L. Ye, S. Hou, H. Xue, Y. Shi, L. Tang, P. Song,J. Gao, *Adv. Sci.* **2024,** 11, 2407295.
